# The acceptability and safety of video triage for ambulance service patients and clinicians during the COVID-19 pandemic

**DOI:** 10.29045/14784726.2021.9.6.2.49

**Published:** 2021-09-01

**Authors:** Fiona Bell, Richard Pilbery, Rob Connell, Dean Fletcher, Tracy Leatherland, Linda Cottrell, Peter Webster

**Affiliations:** Yorkshire Ambulance Service NHS Trust ORCID: https://orcid.org/0000-0003-4503-1903; Yorkshire Ambulance Service NHS Trust ORCID: https://orcid.org/0000-0002-5797-9788; Yorkshire Ambulance Service NHS Trust; Yorkshire Ambulance Service NHS Trust; Yorkshire Ambulance Service NHS Trust; Yorkshire Ambulance Service NHS Trust; Public contributor

**Keywords:** ambulance, EMS, telemedicine, video triage

## Abstract

**Introduction:**

In response to anticipated challenges with urgent and emergency healthcare delivery during the early part of the COVID-19 pandemic, Yorkshire Ambulance Service NHS Trust introduced video technology to supplement remote triage and ‘hear and treat’ consultations as a pilot project in the EOC. We conducted a service evaluation with the aim of investigating patient and staff acceptability of video triage, and the safety of the decision-making process.

**Methods:**

This service evaluation utilised a mixture of routine and bespoke data collection. We sent postal surveys to patients who were recipients of a video triage, and clinicians who were involved in the video triage pilot logged calls they attempted and undertook.

**Results:**

Between 27 March and 25 August 2020, clinicians documented 1073 triage calls. A successful video triage call was achieved in 641 (59.7%) cases. Clinical staff reported that video triage improved clinical assessment and decision making compared to telephone alone, and found the technology accessible for patients. Patients who received a video triage call and responded to the survey (40/201, 19.9%) were also satisfied with the technology and with the care they received. Callers receiving video triage that ended with a disposition of ‘hear and treat’ had a lower rate of re-contacting the service within 24 hours compared to callers that received clinical hub telephone triage alone (16/212, 7.5% vs. 2508/14349, 17.5% respectively).

**Conclusion:**

In this single NHS Ambulance Trust evaluation, the use of video triage for low-acuity calls appeared to be safe, with low rates of re-contact and high levels of patient and clinician satisfaction compared to standard telephone triage. However, video triage is not always appropriate for or acceptable to patients and technical issues were not uncommon.

## Introduction

The emergence of severe acute respiratory syndrome coronavirus 2 (SARS-CoV-2) in China in late 2019 and the subsequent global spread have resulted in a pandemic of coronavirus disease (COVID-19). The protection of healthcare staff has led to the introduction of measures designed to prevent transmission such as reduced face-to-face interactions. This has changed the way that healthcare is delivered across the world, with many healthcare providers introducing remote patient consultations by telephone and video (Greenhalgh et al., 2020).

Prior to the pandemic, ambulance services managed around 6% of their 999 calls with telephone advice only, known as a ‘hear and treat’ response (NHS England, 2020). In order to continue to ensure patient safety was maintained, and to mitigate the impacts of the pandemic on the urgent and emergency care services during the pandemic, Yorkshire Ambulance Service NHS Trust (YAS) began to pilot video consultations for 999 calls managed by the clinical hub within the emergency operations centre (EOC).

Previous research has focused on the use of video consultations for routine outpatient appointments for chronic conditions and there is an absence of evidence of the effectiveness and satisfaction for patients and staff using this technology in an undifferentiated urgent or emergency case load (Greenhalgh et al., 2018). Recent evidence from the United States indicates that telemedicine in urgent hospital presentations provides increased capacity in the healthcare system by reducing face-to-face consultations (Mann et al., 2020). However, it is important to consider the impact of this method of patient assessment and management with respect to the safety of decision making in the pre-hospital setting, patient preference and staff perceptions of the technology (O’Hara et al., 2015).

We aimed to understand the impact of video triage by investigating patient and staff acceptability and the safety of the decision-making process.

## Objectives

Our specific objectives were:

1. To understand the experience of patients who receive video triage as part of an episode of care arising from a 999 call.
2. To understand the experience of ambulance staff who are involved in a patient care episode which used video triage following a 999 call.
3. To understand the outcomes of patients who are offered video triage as determined by:
  - The number of patients who are offered a video consultation but refuse or do not have the technical capability
  - The proportion of 999 calls that are closed as ‘hear and treat’ with and without video triage
  - A comparison of 999 24-hour re-contact rates for video and non-video triaged calls that are closed with ‘hear and treat’ advice.

## Methods

This service evaluation utilised a mixture of routine and bespoke data collection. We sent postal surveys to patients who were recipients of a video triage and had access to activity logs kept by clinicians who were involved in the video triage pilot.

### Setting

YAS provides 24-hour emergency and healthcare services for the county of Yorkshire in the north of England. The county has a population of approximately five million, spread over almost 6000 square miles of varied terrain, including isolated moors and dales, coastline and heavily populated urban areas. In 2019/2020, the emergency operations centre (EOC) in YAS received more than 1,054,575 calls and responded to 847,949 incidents by either sending clinicians to scene or providing assessment and advice over the telephone.

Within the EOC at YAS, there is a clinical hub staffed by paramedics and nurses (referred to as senior clinical advisors, SCAs) who can provide telephone triage services to all categories of incident responded to by the service. In addition, they may provide telephone support to operational resources on-scene, to assist with decision making such as whether to convey patients to hospital. Due to the pandemic, a number of clinical hub and front-line operational staff were required to shield for 12 weeks. Implementation of remote telephone triage and consultation enabled these staff to continue working and this was supplemented by the addition of the video triage pilot.

### Patients

Calls eligible for video triage were initially restricted to adults aged 18 to 65 years that had been triaged as suitable for the clinical hub to review and manage. However, as the pilot continued, restrictions on calls were relaxed and extended to include category 2 and 3 calls and all age ranges. Patients under the age of 16 years were assessed with the consent of their parent or guardian.

### Data sources

Video consultations, using either AcuRX™ or GoodSAM™, commenced on 23 March 2020 and information about the call was recorded by clinicians participating in the pilot. Clinicians also recorded their perception of the experience of video triage for patients and themselves, to determine acceptability and impact on the assessment process (Supplementary 1). This enhanced data collection commenced on 9 May 2020 and continued until the end of the pilot.

To capture the patient experience, a YAS patient research ambassador was consulted on the creation of a brief postal survey (Supplementary 2), which was sent to recipients of a video consultation. Surveys were sent to patients between 11 June and 13 August 2020.

In the absence of any validated patient-reported experience measures tailored to urgent and emergency care (UEC), we developed our own based on published indicators of ambulance service performance that are focused on patient outcomes: access; acceptability; decisions (e.g. to leave at home); satisfaction; professionalism; and holistic care (e.g. physical, social, emotional needs) (National Quality Forum, 2017; Turner et al., 2019).

The patient survey consisted of seven Likert-style questions, although survey respondents also had the option to make free-text comments. The free-text comments were stratified into broad themes and the most commonly recurring themes reported, with examples of each provided.

To determine the 24-hour re-contact rate, data were collected from the YAS 999 Computer Aided Dispatch (CAD) system for all video triage calls. A comparison group, comprised of calls initially triaged as category 5 which were managed by the clinical hub (since these calls could reasonably be assumed to have a disposition of ‘hear and treat’), was also obtained. A re-contact was said to have occurred if a patient with the same NHS number, or same name, age, sex and incident location, called 999 in the 24 hours after the first call was recorded as being closed.

### Statistical methods

No formal sample was calculated for this service evaluation and the analysis plan was predominantly descriptive. Likert-style question responses analysed using horizontal diverging stacked bar charts and clinician call activity were reported using counts and proportions stratified by video calling application (GoodSAM or accuRx). A sankey diagram was utilised to demonstrate change in initial and final triage category, and final call outcome.

The differences in re-contact rates of category 5 calls between video calls and YAS-wide performance were reported as proportions, as were the rates of category 5 calls resulting in a ‘hear and treat’ outcome.

## Results

### Summary of patient responses

**Figure 1:**
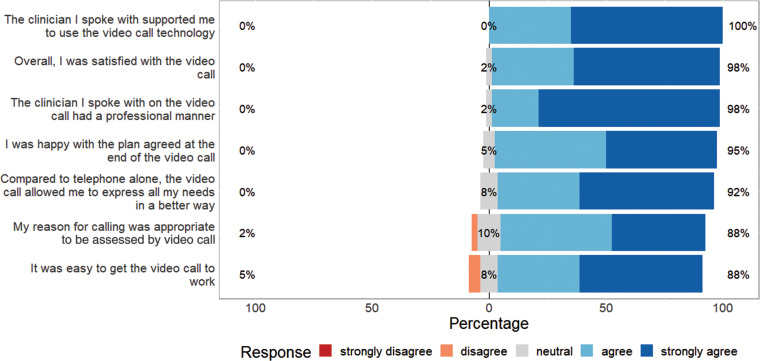
Figure 1. Patient responses to video triage call survey.

Between 11 June and 13 August 2020, postal surveys were sent to 201 patients who were the recipient of a video triage call. Of these, 40/201 (19.9%) were returned prior to the evaluation closing. Patients that responded viewed the technology, the ambulance staff and the care planning favourably. In addition to the specific Likert questions, there was an opportunity for respondents to enter a free-text comment, which was utilised in 28/45 (62.2%) cases (Figure 1 and Supplementary 3). The majority of comments praised the clinicians involved in their care, both over the phone and in person:

The lady I spoke to was extremely helpful and professional. I really appreciate the help she gave me regarding my six year old son. Many thanks. Second-to-none service.

The ambulance staff were absolute first class, cannot fault them in any way, a credit to the service.

Another common thread related to the benefits of video calling over telephone-only triage, for example:

Thank you. It was a very helpful call in an emergency situation and was more reassuring than a telephone call.

Although my technology skills are mid-range at best, I found the video call easy to manage. It also allowed the person dealing with my problem to see exactly what difficulties I was experiencing and to deal with them in the most appropriate way.

However, four respondents highlighted technical issues with the video triage service:

Only issue was connection / signal, very hard to hear clearly. The staff were faultless.

The video kept freezing and the sound was very bad so the call did not achieve anything. So was not a satisfactory outcome.

### Summary of clinician data

**Figure 2:**
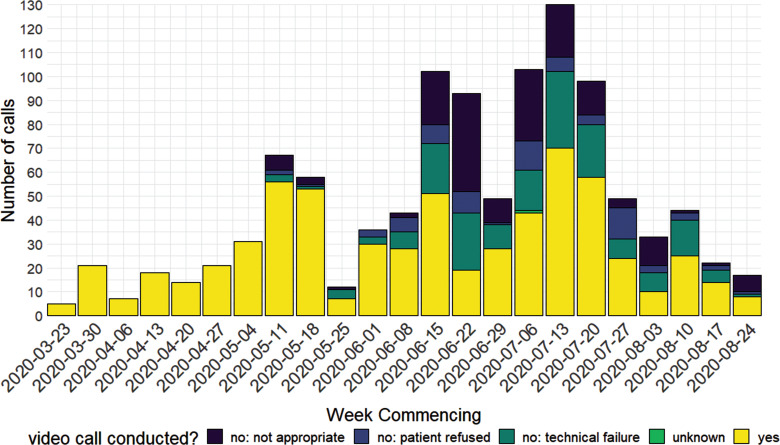
Figure 2. Calls per week, stratified by video call conducted.

Between 27 March and 25 August 2020, clinicians documented 1073 triage calls. A video call was conducted in 641 (59.7%) cases (Table 1). Video call activity appeared to peak in the middle of May and then again in July. The addition of further trained staff as video users did not appear to increase the volume of successful video calls (Figure 2).

**Table 1.** Triage calls undertaken by clinicians stratified by initial triage category.

	Level	Overall	1	2	3	4	5	Unknown
n		1073	11	40	102	17	850	53
Month (%)	Mar	14 (1.3)	0 (0.0)	3 (7.5)	3 (2.9)	0 (0.0)	8 (0.9)	0 (0.0)
	Apr	59 (5.5)	1 (9.1)	5 (12.5)	15 (14.7)	1 (5.9)	26 (3.1)	11 (20.8)
	May	181 (16.9)	0 (0.0)	1 (2.5)	13 (12.7)	1 (5.9)	126 (14.8)	40 (75.5)
	Jun	290 (27.0)	0 (0.0)	8 (20.0)	29 (28.4)	5 (29.4)	248 (29.2)	0 (0.0)
	Jul	403 (37.6)	9 (81.8)	19 (47.5)	35 (34.3)	8 (47.1)	330 (38.8)	2 (3.8)
	Aug	126 (11.7)	1 (9.1)	4 (10.0)	7 (6.9)	2 (11.8)	112 (13.2)	0 (0.0)
CBU (%)	North & East Yorkshire CBU	264 (24.6)	4 (36.4)	6 (15.0)	24 (23.5)	3 (17.6)	213 (25.1)	14 (26.4)
	South Yorkshire CBU	332 (30.9)	4 (36.4)	12 (30.0)	35 (34.3)	3 (17.6)	264 (31.1)	14 (26.4)
	West Yorkshire CBU	471 (43.9)	3 (27.3)	21 (52.5)	43 (42.2)	11 (64.7)	368 (43.3)	25 (47.2)
	N/A	6 (0.6)	0 (0.0)	1 (2.5)	0 (0.0)	0 (0.0)	5 (0.6)	0 (0.0)
Video (%)	Accurx™	90 (8.4)	0 (0.0)	1 (2.5)	7 (6.9)	1 (5.9)	77 (9.1)	4 (7.5)
	Goodsam™	927 (86.4)	11 (100.0)	38 (95.0)	90 (88.2)	15 (88.2)	739 (86.9)	34 (64.2)
	Unknown	56 (5.2)	0 (0.0)	1 (2.5)	5 (4.9)	1 (5.9)	34 (4.0)	15 (28.3)
Final triage category (%)	1	2 (0.2)	1 (9.1)	0 (0.0)	0 (0.0)	0 (0.0)	0 (0.0)	1 (1.9)
	2	264 (24.6)	8 (72.7)	30 (75.0)	17 (16.7)	4 (23.5)	190 (22.4)	15 (28.3)
	3	348 (32.4)	2 (18.2)	2 (5.0)	59 (57.8)	1 (5.9)	273 (32.1)	11 (20.8)
	4	84 (7.8)	0 (0.0)	4 (10.0)	3 (2.9)	11 (64.7)	64 (7.5)	2 (3.8)
	5	375 (34.9)	0 (0.0)	4 (10.0)	23 (22.5)	1 (5.9)	323 (38.0)	24 (45.3)
Age range (%)	<5 years	39 (3.6)	0 (0.0)	2 (5.0)	2 (2.0)	0 (0.0)	34 (4.0)	1 (1.9)
	5–12 years	27 (2.5)	0 (0.0)	3 (7.5)	0 (0.0)	0 (0.0)	21 (2.5)	3 (5.7)
	13–17 years	21 (2.0)	0 (0.0)	0 (0.0)	3 (2.9)	0 (0.0)	17 (2.0)	1 (1.9)
	18–64 years	488 (45.5)	8 (72.7)	32 (80.0)	59 (57.8)	15 (88.2)	333 (39.2)	41 (77.4)
	65+ years	488 (45.5)	3 (27.3)	1 (2.5)	38 (37.3)	2 (11.8)	437 (51.4)	7 (13.2)
	N/A	10 (0.9)	0 (0.0)	2 (5.0)	0 (0.0)	0 (0.0)	8 (0.9)	0 (0.0)
Sex (%)	Female	565 (52.7)	7 (63.6)	22 (55.0)	57 (55.9)	13 (76.5)	440 (51.8)	26 (49.1)
	Male	498 (46.4)	4 (36.4)	15 (37.5)	44 (43.1)	4 (23.5)	405 (47.6)	26 (49.1)
	Unknown	8 (0.7)	0 (0.0)	3 (7.5)	0 (0.0)	0 (0.0)	4 (0.5)	1 (1.9)
	N/A	2 (0.2)	0 (0.0)	0 (0.0)	1 (1.0)	0 (0.0)	1 (0.1)	0 (0.0)
Video call conducted? (%)	No: not appropriate	176 (16.4)	1 (9.1)	5 (12.5)	9 (8.8)	1 (5.9)	160 (18.8)	0 (0.0)
	No: patient refused	74 (6.9)	1 (9.1)	6 (15.0)	12 (11.8)	3 (17.6)	51 (6.0)	1 (1.9)
	No: technical failure	181 (16.9)	3 (27.3)	4 (10.0)	20 (19.6)	4 (23.5)	149 (17.5)	1 (1.9)
	Unknown	1 (0.1)	0 (0.0)	0 (0.0)	0 (0.0)	0 (0.0)	1 (0.1)	0 (0.0)
	Yes	641 (59.7)	6 (54.5)	25 (62.5)	61 (59.8)	9 (52.9)	489 (57.5)	51 (96.2)
Technical issue during call? (%)	No	728 (67.8)	9 (81.8)	30 (75.0)	67 (65.7)	10 (58.8)	584 (68.7)	28 (52.8)
	Yes	345 (32.2)	2 (18.2)	10 (25.0)	35 (34.3)	7 (41.2)	266 (31.3)	25 (47.2)
AMPDS chief complaint (%)	Abdominal pain/problems	67 (6.2)	0 (0.0)	0 (0.0)	0 (0.0)	10 (58.8)	57 (6.7)	0 (0.0)
	Allergies	8 (0.7)	2 (18.2)	0 (0.0)	0 (0.0)	0 (0.0)	6 (0.7)	0 (0.0)
	Assault	6 (0.6)	0 (0.0)	1 (2.5)	1 (1.0)	0 (0.0)	4 (0.5)	0 (0.0)
	Back pain	40 (3.7)	0 (0.0)	0 (0.0)	1 (1.0)	0 (0.0)	37 (4.4)	2 (3.8)
	Breathing problems	16 (1.5)	1 (9.1)	1 (2.5)	1 (1.0)	0 (0.0)	12 (1.4)	1 (1.9)
	Diabetic problems	9 (0.8)	0 (0.0)	1 (2.5)	0 (0.0)	0 (0.0)	8 (0.9)	0 (0.0)
	Falls	379 (35.3)	1 (9.1)	1 (2.5)	21 (20.6)	0 (0.0)	355 (41.8)	1 (1.9)
	Haemorrhage/lacerations	21 (2.0)	3 (27.3)	4 (10.0)	0 (0.0)	0 (0.0)	14 (1.6)	0 (0.0)
	Overdose/poisoning	6 (0.6)	1 (9.1)	1 (2.5)	1 (1.0)	0 (0.0)	3 (0.4)	0 (0.0)
	Pandemic	105 (9.8)	0 (0.0)	0 (0.0)	9 (8.8)	0 (0.0)	62 (7.3)	34 (64.2)
	Sick person	48 (4.5)	0 (0.0)	0 (0.0)	0 (0.0)	0 (0.0)	48 (5.6)	0 (0.0)
	Stroke/TIA	25 (2.3)	0 (0.0)	0 (0.0)	3 (2.9)	0 (0.0)	22 (2.6)	0 (0.0)
	Traumatic injuries	114 (10.6)	0 (0.0)	6 (15.0)	0 (0.0)	0 (0.0)	107 (12.6)	1 (1.9)
	Unconscious	25 (2.3)	0 (0.0)	1 (2.5)	8 (7.8)	0 (0.0)	16 (1.9)	0 (0.0)
	Other	32 (3.0)	1 (9.1)	1 (2.5)	4 (3.9)	1 (5.9)	24 (2.8)	1 (1.9)
	N/A	172 (16.0)	2 (18.2)	23 (57.5)	53 (52.0)	6 (35.3)	75 (8.8)	13 (24.5)
Call outcome (%)	Hear and treat	424 (39.5)	2 (18.2)	24 (60.0)	42 (41.2)	10 (58.8)	320 (37.6)	26 (49.1)
	Unknown	33 (3.1)	0 (0.0)	0 (0.0)	4 (3.9)	1 (5.9)	24 (2.8)	4 (7.5)
	See, treat and convey	418 (39.0)	8 (72.7)	13 (32.5)	33 (32.4)	5 (29.4)	345 (40.6)	14 (26.4)
	See, treat and refer	198 (18.5)	1 (9.1)	3 (7.5)	23 (22.5)	1 (5.9)	161 (18.9)	9 (17.0)

**Figure 3:**
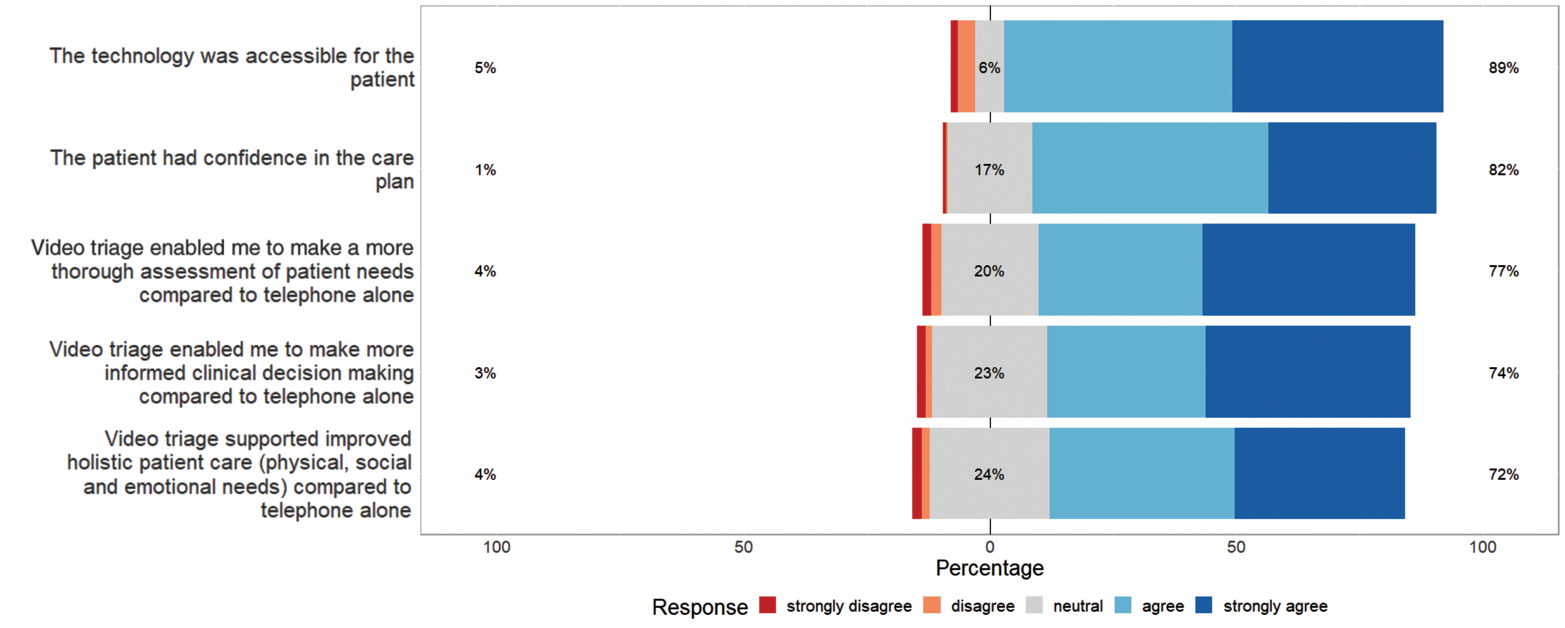
Figure 3. Likert responses by clinicians relating to video triage call.

There were up to 713 responses from clinicians in relation to the Likert-style questions, with not all questions answered for every video call. Clinicians were only slightly more equivocal than patients about the impact of video triage in terms of improving their assessment and care of patients and the accessibility of the technology (Figure 3).

**Figure 4:**
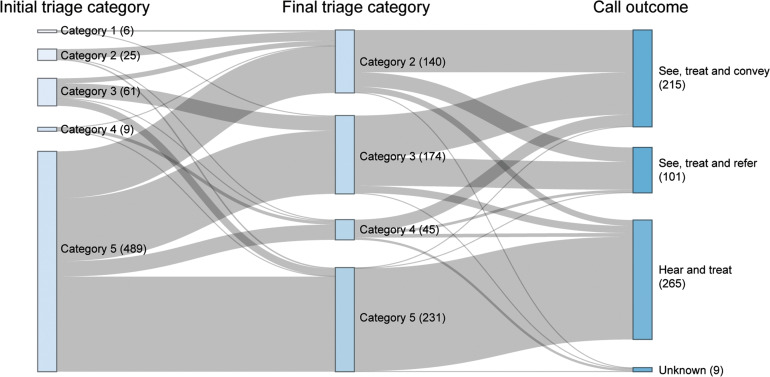
Figure 4. Relationship of initial and final triage category and call outcome for successful video triage calls (Note: 51 cases did not have an initial triage category reported and have been removed).

The majority of calls selected for video triage by clinicians were initially triaged as category 5. However, as Figure 4 shows, over half of calls were categorised to a higher priority by the clinician after the video consultation.

**Figure 5:**
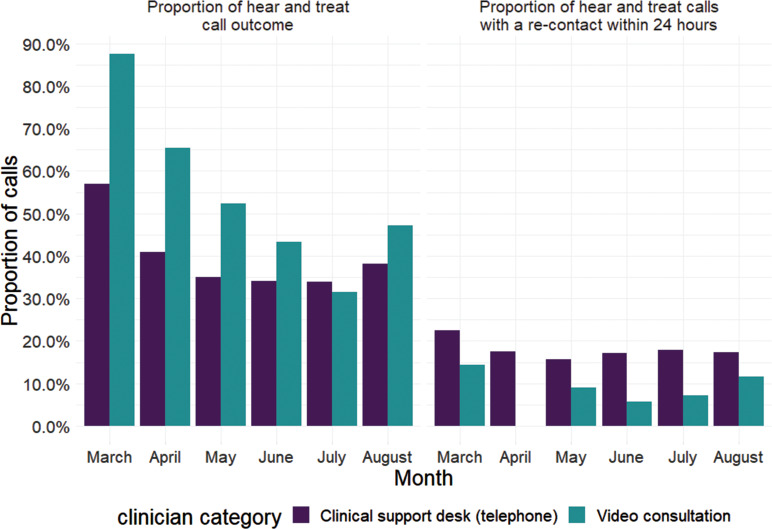
Figure 5. Comparison of clinical hub and video triage category 5 calls.

An analysis of category 5 calls shows that a higher proportion of calls that were initially triaged as category 5 remained so when a video triage was conducted compared to traditional telephone triage conducted by the clinical hub. Similarly, a higher proportion of calls had an outcome of ‘hear and treat’ when video triage was utilised (Table 2 and Figure 5). Re-contact rates within 24 hours of initial call for the subset of calls with a ‘hear and treat’ outcome were 2508/14349 (17.5%) for clinical hub calls and 16/212 (7.5%) for the video calls during the same period.

**Table 2.** Comparison of category 5 call outcome when managed by clinicians utilising video triage compared to regular telephone triage conducted by clinical hub clinicians.

	Level	Overall	Clinical hub (telephone)	Video consultation
n		39148	38,659	489
Final triage category (%)	1	55 (0.1)	55 (0.1)	0 (0.0)
	2	5715 (14.6)	5611 (14.5)	104 (21.3)
	3	16140 (41.2)	16,000 (41.4)	140 (28.6)
	4	2702 (6.9)	2668 (6.9)	34 (7.0)
	5	14536 (37.1)	14,325 (37.1)	211 (43.1)
Call outcome (%)	Hear and treat	14561 (37.2)	14,349 (37.1)	212 (43.4)
	Unknown	2403 (6.1)	2396 (6.2)	7 (1.4)
	See, treat and convey	11,804 (30.2)	11,621 (30.1)	183 (37.4)
	See, treat and refer	10,380 (26.5)	10,293 (26.6)	87 (17.8)

## Discussion

The pandemic has seen a rapid expansion of telemedicine in the NHS, specifically video consultations. This has been achieved by centrally funded procurement, championing of the technology by NHSX and flexibility of conformance with information governance rules and legislation (NHSx, 2020a, 2020b). While several ambulance services are utilising video triage (London Ambulance Service NHS Trust, 2019; North East Ambulance Service NHS Trust, n.d.), to date no published studies exist with respect to the clinical and economic benefits of the technology.

Randomised controlled trials in the use of video triage for clinical consultations have generally been underpowered and limited to carefully selected groups of patients in secondary care (Greenhalgh et al., 2020).

Studies relating specifically to the ambulance service are small and while results are encouraging in terms of feasibility, the studies do not map out the economic benefit of such interventions and generally raise concerns about technical issues and/or acceptability of the technology by clinical staff (Johansson et al., 2019; Linderoth et al., 2019; Rogers et al., 2017). Clinicians in this evaluation noted that video calling could not take place due to technology failures in 16.9% of cases. It is pertinent to note that in a recent systematic review of telemedicine systems in ambulances, none of the included studies considered acceptability by patients (Rogers et al., 2017), and patient refusal was identified in 6.9% of cases here.

From the limited number of patient surveys returned, it appears that patients in our evaluation are satisfied with the use of video call technology. Clinicians reported that, where a call took place, video calling was acceptable compared to traditional methods of triage. In over 70% of calls, video consultation was perceived to be superior to telephone alone for patient assessment, decision making and patient care. Clinicians did however view video calls overall less favourably than patients.

Unsurprisingly, the majority of calls (850/1073, 79.2%) considered for video triage call were initially triaged as category 5, although higher categories of calls were answered as the pilot progressed and the eligibility rules were relaxed. There was some movement between initial and final triage category, with 2.1% of calls initially triaged as category 3 being downgraded to category 5, for example. However, conversely, substantial proportions of calls initially categorised as category 5 were upgraded to category 2 (190/850, 22.4%) or 3 (273/850, 32.1%).

While elsewhere, it appears that younger patients may be more likely to utilise the recent push for remote consultations (Mann et al., 2020), our evaluation was more representative of the typical distribution of ambulance service patients, with an even split between adults over and under the age of 65 years. The difference in age demographic may be related to the patient types included in each dataset, in particular that the majority of YAS cases were category 5 calls which disproportionally include older falls patients (Table 1).

Re-contact rate reporting was phased out of the Ambulance Quality Indicators in April 2018 although the last month in which all services were still reporting re-contact within 24 hours was July 2017 (NHS England, 2020). Data from this month show that the national (England) mean re-contact rate was 6.7%, although there was considerable variation between ambulance services (0.8% to 15.0%), perhaps suggesting that services with the lowest re-contact rates were not able to reliably detect re-contacts.

It is difficult to compare historic re-contact rates with those occurring during a pandemic, but the re-contact rate for calls closed with ‘hear and treat’ advice by the clinical hub was 17.5%. Video triaged calls had a lower re-contact rate of 7.5%, although this is still higher than the aforementioned English mean re-contact rate.

## Limitations

This project is a real-world evaluation and carried out in a single NHS Ambulance Trust so the results must be interpreted with caution. The patient and clinician satisfaction measures have not been validated and therefore may not be the most appropriate measures for this evaluation, but they provide a starting point for assessing the delivery of patient-centred care via video. In addition, the response to the patient survey was low and not all recipients of a video triage call received a survey, limiting the generalisability of these results.

The source data for the video triage calls were self-reporting by participating clinicians and it is possible that cases were missed and affected by the clinician’s perception of calls that were considered appropriate for video consultation.

Previous models have used the definition of patients attending ED or being admitted to hospital within 3 days of contact as being an incorrect ‘hear and treat’ or ‘see and treat’ response (Turner et al., 2019). We took a pragmatic decision to limit re-call to 24 hours since the difference between telephone and video triage in terms of re-contact was not anticipated to be significant and meant the data were provided sooner. However, poor recording of patient demographics required for matching callers could have resulted in some re-contacts not being detected.

## Conclusion

In this single NHS Ambulance Trust evaluation, the use of video triage for low acuity calls appeared to be safe, with low rates of re-contact and high levels of patient and clinician satisfaction compared to standard telephone triage. However, video triage is not always appropriate or acceptable to patients and technical issues were not uncommon.

## Acknowledgements

This work uses data provided by patients and collected by the NHS as part of their care and support. The authors would also like to thank the clinicians of Yorkshire Ambulance Service who participated in the pilot and provided additional data to support this evaluation. Thanks also to Dr Fiona Sampson who kindly commented on a draft of this manuscript.

## Author contributions

FB, RC and DF conceived and designed the evaluation, RP, TL, LC and PW designed the data collection tools. RP undertook the analysis. All authors drafted the manuscript and contributed substantially to its revision. FB acts as the guarantor for this article.

## Conflict of interest

RP is on the editorial board of the BPJ but had no part in reviewing or considering this article. None of the other authors declare any conflicts of interest with respect to this service evaluation.

## Data sharing statement

The data for this evaluation are available on reasonable request.

## Ethics

Formal Research Ethics Committee approval was not required for this work as it is a service evaluation. Approval for the evaluation to be conducted and confirmation that NHS REC was not required were obtained from the research and development department at Yorkshire Ambulance Service.

## Funding

None.
